# Effect of TRPV4-p38 MAPK Pathway on Neuropathic Pain in Rats with Chronic Compression of the Dorsal Root Ganglion

**DOI:** 10.1155/2016/6978923

**Published:** 2016-06-05

**Authors:** Yu-Juan Qu, Xiao Zhang, Zhen-Zhen Fan, Juan Huai, Yong-Bo Teng, Yang Zhang, Shou-Wei Yue

**Affiliations:** ^1^Department of Physical Medicine and Rehabilitation, Qilu Hospital of Shandong University, No. 107 Wenhuaxi Road, Jinan, Shandong 250012, China; ^2^Department of Physical Medicine and Rehabilitation, Jinan Military General Hospital, No. 25, Shifan Road, Jinan, Shandong 250031, China

## Abstract

The aim of this study was to investigate the relationships among TRPV4, p38, and neuropathic pain in a rat model of chronic compression of the dorsal root ganglion. Mechanical allodynia appeared after CCD surgery, enhanced via the intrathecal injection of 4*α*-phorbol 12,13-didecanoate (4*α*-PDD, an agonist of TRPV4) and anisomycin (an agonist of p38), but was suppressed by Ruthenium Red (RR, an inhibitor of TRPV4) and SB203580 (an inhibitor of p38). The protein expressions of p38 and P-p38 were upregulated by 4*α*-PDD and anisomycin injection but reduced by RR and SB203580. Moreover, TRPV4 was upregulated by 4*α*-PDD and SB203580 and downregulated by RR and anisomycin. In DRG tissues, the numbers of TRPV4- or p38-positive small neurons were significantly changed in CCD rats, increased by the agonists, and decreased by the inhibitors. The amplitudes of ectopic discharges were increased by 4*α*-PDD and anisomycin but decreased by RR and SB203580. Collectively, these results support the link between TRPV4 and p38 and their intermediary role for neuropathic pain in rats with chronic compression of the dorsal root ganglion.

## 1. Introduction

After tissue injury and inflammation, the sensory signals from the primary sensory neurons to the spinal dorsal horn change significantly, ultimately leading to the development of chronic pain [1]. Abnormal pain manifestations including allodynia, hyperalgesia, and spontaneous pain episodes are believed to partially result from plastic phenomena in the spinal sensory system [2, 3]. These manifestations, known as neuropathic pain, are caused by the primary injury or functional disability of the nervous system. Radicular neuralgia is the most common form of neuropathic pain; it occurs when the radix spinalis or the dorsal root ganglia (DRGs) are stimulated by harmful factors (e.g., the protrusion of a lumbar intervertebral disc, lumbar spinal stenosis, spinal cord tumor compression, and certain inflammatory substances). They then become excited to create and transmit neuropathic pain signals [4]. Our studies have used chronic compression of the DRGs (CCD) as a typical model of neuropathic pain that demonstrates spontaneous pain, hyperalgesia, and allodynia and is accompanied by increased spontaneous discharges of neurons as well as decreased action potentials and electric current thresholds [5].

When the physiopathological mechanism of inflammatory pain was studied in patients with amputation neuroma, spinal cord injury, or other models of neuropathic pain, mitogen-activated protein kinases (MAPKs, e.g., ERK, JNK, and p38) played a critical role. The phosphorylated forms of these kinases maintain and enlarge the pain signal from the peripheral nociceptors or DRGs by modifying proteins posttranslationally and regulating the transcription of critical genes. The local injection of MAPK inhibitors significantly depresses thermal and mechanical hyperalgesia [6–8]. p38 is an important MAPK involved in inflammation-induced pain and is activated by a variety of acute stimuli, including the intraplantar injection of formalin and the intrathecal injection of substance P [9]. Furthermore, p38 reduces pain by inhibiting p38 phosphorylation through decreased TNF-*α* [10]. Pregabalin reduces the nociceptive reaction in a zymosan-induced inflammatory pain model by inhibiting the phosphorylation of MAPKs [11]. Considerable evidence indicates that p38 is an important mediator of the NF-*κ*B pathway, which might be inhibited by SB203580 through the NF-*κ*B pathway [12, 13]. Thus, the p38 and the NF-*κ*B pathway are likely key contributors in inflammation [14–16].

Transient receptor potential (TRP) channels are Ca^2+^-permeable cation channels that play important roles in sensory function. These channels can be activated by a variety of stimuli, including mechanical and osmotic stress, thermal stimuli, and chemical signals [17, 18]. TRP vanilloid receptor 4 (TRPV4) is a member of the vanilloid subfamily of TRP channels, and accumulating experimental evidence over the past decade has increasingly clarified its role in pain signal transduction [19–27]. Our preliminary studies showed that TRPV4 participates in the mechanisms associated with the high excitability of neurons, hyperalgesia, and allodynia after CCD surgery. Moreover, TRPV4 antisense oligodeoxynucleotides partially restore the decreased mechanical withdrawal threshold after CCD surgery without influencing the basal threshold [28]. These antisense oligodeoxynucleotides exert their effect through the TRPV4-NO pathway mediated by NF-*κ*B [29]. TRPV4 ion channels can be induced via a hypotonic solution and 4*α*-PDD; furthermore, they open with increased inward current, leading to a peak of intracellular calcium. This increasing intracellular calcium activates p38 by phosphorylating it into a biologically functional form, mediating mechanical allodynia and inflammation-induced hyperalgesia [30]. Capsazepine, the antagonist of another TRP family member, TRPV1, activates p38, JNK, and ERK1/2 dose-dependently and upregulates the death receptors (DRs) through activated JNK [31]. No direct evidence has been reported with regard to the relationship between TRPV4 and p38. Thus, we formed the following hypothesis: activated and upregulated TRPV4 in the DRG neurons of a CCD model mediates the formation and transmission of neuropathic pain through the alternation of p38.

Spontaneous pain is likely related to the abnormal electrical activity and activated TRPV4 ion channels that mediate calcium and sodium internal flow, creating ectopic discharges [32]. The nerve fibers involved in ectopic discharges include A and C fibers; A fibers are thin, have a myelin sheath, and mediate stabbing pain, whereas C fibers are thick, do not have a myelin sheath, and mediate causalgia. C fibers root from small neurons in the DRGs and primarily conduct slow pain.

We investigated the expression of TRPV4 and p38 after CCD surgery and then determined whether agonizing or inhibiting TRPV4 and p38 changed the expression and location pattern of those proteins. Finally, we examined the effects of those agonists or inhibitors on the development of mechanical allodynia and ectopic discharges.

## 2. Materials and Methods

### 2.1. Materials

#### 2.1.1. Experimental Animals

Adult SPF male Wistar rats with weights ranging from 180 g to 200 g, provided by the Experimental Animal Center of Shandong University, were housed in a room with pathogen-free air, at 20 ± 2°C, two per cage, on a 12 h light/dark cycle. Water and food were available ad libitum. The animals were allowed 7 days to habituate to their housing prior to manipulation and 30 min to habituate to the experimentation environment before each behavioral study was performed. The Animal Care and Use Committee of Shandong University approved all experimental procedures.

#### 2.1.2. Reagents

Four days after CCD surgery, the TRPV4 inhibitor Ruthenium Red (RR, Sigma, Germany), the TRPV4 agonist 4*α*-PDD (CST, USA), the p38 inhibitor SB203580 (CST, USA), or the p38 agonist anisomycin (CST, USA) was given to the experimental groups at the recommended concentrations via intrathecal injection.

### 2.2. CCD Model

After anesthetizing the rats with 10% chloral hydrate (300 mg/100 g body weight, i.p.), the animals were shaved and sterilized. Then, their skin was cut between the bilateral spina iliac, extending upward approximately 2 cm. Next, the deep fascia and muscle were longitudinally cut upward approximately 2 cm from the end of the right fascia triangle; the tail levator was pushed aside so that the mastoid of L4 and L5 was visible. After separating the covering muscles, the outer intervertebral foramen of L4 and L5 was revealed; then, sterile L-shaped steel bars (diameter = 0.63 mm) were inserted into the intervertebral foramen of L4 and L5 at a 30° oblique angle with the spinal column, keeping the other end of the steel bar out of the intervertebral foramen. After the operation, the incisions were washed with normal saline; then, the muscle, fascia, and skin were sutured in sequence, and penicillin was given intraperitoneally to prevent infection. The rats that developed autophagy, sensory deficiency, or disability were eliminated from analysis.

### 2.3. Behavioral Testing

Walk gait pattern was assessed as an index of motor function. A score of 1 indicates a normal gait, without foot deformities; a score of 2 indicates a normal gait with obvious foot deformities; a score of 3 indicates a slight gait disturbance with foot-drop; and a score of 4 indicates a serious gait disturbance with myasthenia. Only rats scoring 1 were used for the following experimental procedures.

The behavioral testing was performed with regard to the ipsilateral hind paw of the animals prior to surgery as well as on postoperative days 2, 4, 6, 10, 14, and 28. The effects of the inhibitor or agonist on the CCD-induced allodynia were tested between 0.5-h and 8-h after injection. The paw withdrawal mechanical threshold (PWMT) was evaluated using a BME-404 Mechanical Analgesia Tester (Chinese Academy of Medical Sciences, CAMS, Beijing, China). A probe was pressed against the lateral plantar surface of the hind paws with sufficient force. A positive response was noted when the paw immediately withdrew. The procedure was repeated five times at least 5 min apart, and the average value was used as a variable.

### 2.4. Western Blot Analysis

The L4 and L5 ganglia from the operated side were harvested quickly and carefully. Protein samples of the DRGs were prepared on ice. Then, the sample of total protein was separated by 5% and 10% SDS-PAGE. Proteins were transferred to polyvinylidene fluoride membranes. The membranes were incubated in 5% milk for 2 h at room temperature. Then, the membranes were incubated with the primary antibody at 4°C overnight followed by horseradish peroxidase- (HRP-) conjugated secondary antibodies for 1 h. The signal was detected using the Immobilon*™* Western Chemiluminescent HRP Substrate. The primary antibodies were rabbit anti-TRPV4 polyclonal antibody (1 : 800, Abcam, Cambridge, UK), rabbit anti-p38 polyclonal antibody (1 : 200, CST, USA), and rabbit anti-P-p38 polyclonal antibody (1 : 1,000, CST, USA), whereas the second antibody was a goat anti-rabbit antibody (1 : 8,000, Zhongshan Goldenbridge, Beijing, China). The protein bands were visualized using a FluoroChem 9900 Imaging System (USA), and the bands' intensity was quantified with the Quantity One software and normalized to *β*-tubulin (1 : 1,000, CST, USA).

### 2.5. Immunohistochemistry

After the behavioral and pain tests, the rats were deeply anesthetized with 5% isoflurane and perfused transcardially with cold normal saline followed by a fixative containing 4% paraformaldehyde and 0.2% picric acid in 0.1 M phosphate-buffered saline (PBS, pH 6.9). Ipsilateral lumbar L4-L5 DRGs were removed rapidly after perfusion, postfixed in the same fixative overnight at 4°C, and then dehydrated and paraffin-infused. A series of 4-*μ*m paraffin sections were cut using a rotary microtome. The sections were heated at 65°C for at least 2 h and then deparaffinized. Antigen retrieval was accomplished with citrate buffer in a microwave oven at 92–98°C for 15–20 min. The sections were washed in PBS and then incubated separately in rabbit anti-TRPV4 polyclonal antibody (1 : 200, Abcam, Cambridge, UK), rabbit anti-p38 polyclonal antibody (1 : 50, CST, USA), and rabbit anti-P-p38 polyclonal antibody (1 : 100, CST, USA) at 4°C overnight. The sections were incubated using a specific secondary antibody for 2 h at room temperature. DAB substrate solution and hematoxylin were used to develop the color. Labeled sections were examined under a Leica Quantimet 550 DMRXA automated research microscope (GER) and analyzed using IPP.6.

### 2.6. Ectopic Spontaneous Discharge Recording

Four days after CCD surgery, rats without autophagy, sensory deficiency, or disability were selected. The animals were anesthetized with 10% chloral hydrate (300 mg/100 g body weight, i.p.). Their skin was cut, their muscles were moved, and the vertebral plate of their L2–L6 was removed carefully to avoid spinal cord or nerve injury. The steel bars were removed from the DRGs, the peripheral nerves were cut off approximately 10 mm from the L4 and L5 DRGs to block signaling from the peripheral receptors, and the communicating branches of L4 and L5 were cut to block signals from other segments. A bath around the incision was built and filled with 35°C–37°C manual cerebrospinal fluid, containing 150 mmol/L NaCl, 1 mmol/L MgCl_2_, 2 mmol/L CaCl_2_, 10 mmol/L glucose, and 5 mmol/L Tris at pH 7.4. The central ends of L4 and L5 were covered with 35°C–37°C medicinal liquid paraffin. The nerves were divided into several 30 to 50-*μ*m fiber bundles under a microscope and hung on the recording electrode separately, with the reference electrode inserted into the skin nearby. Discharge signals were recorded using a BL-420E+ biological and functional experimental system (Taimeng Science and Technology Ltd., Chengdu, China).

### 2.7. Statistical Analyses

All calculations and statistical analyses were performed using Prism 5.0 (GraphPad Software, San Diego, CA, USA). Data values were expressed as means ± SEM. *P* values < 0.05 were considered significant. The analyses were performed using one-way (with Tukey* post hoc* tests) and two-way ANOVAs.

## 3. Results

### 3.1. Changes in PWMT in CCD Rats after the Injection of Agonists and Inhibitors

**Figure 1:**
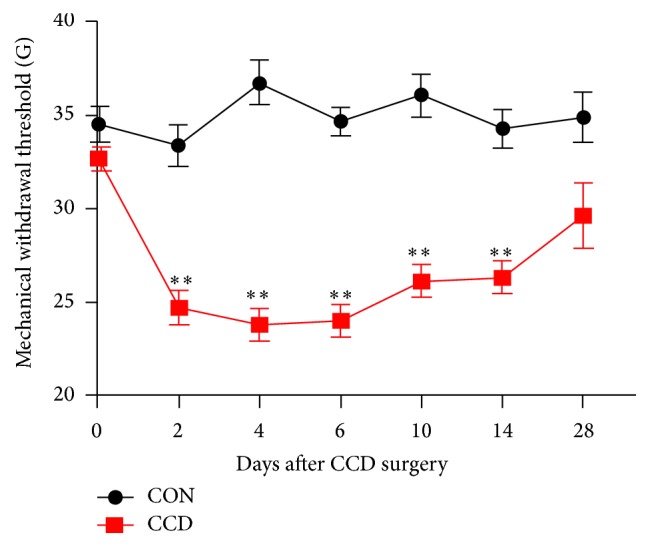
Alternations of PWMT in CCD rats. Mechanical allodynia of the ipsilateral hind paws induced by CCD (*n* = 8 in each group); ^*∗∗*^ *P* < 0.01 compared with controls.

**Figure 2:**
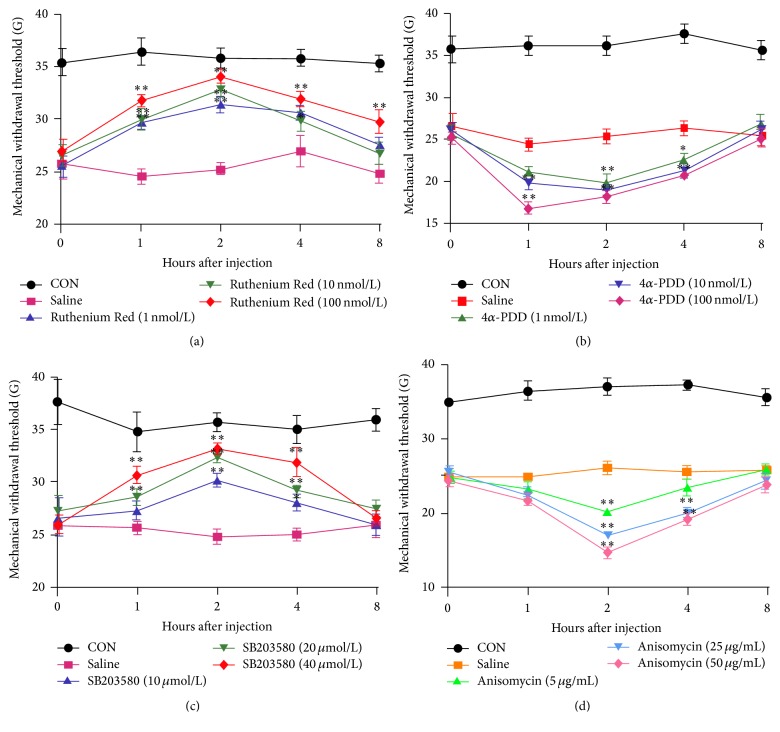
The effects of the reagents on CCD-induced mechanical allodynia. (a–d) The PWMTs of CCD rats (4 days after operation) 1, 2, 4, and 8 h after RR, 4*α*-PDD, SB203580, and anisomycin intrathecal injections (*n* = 6; the data are expressed as means ± SEMs); ^*∗*^ *P* < 0.05 and ^*∗∗*^ *P* < 0.01 compared ipsilaterally with the saline group; one-way ANOVA followed by Tukey's* post hoc* test.

Via behavioral testing, we first determined the mechanical allodynia regulation of the ipsilateral hind paw compared with controls. PWMT significantly decreased from the second day after CCD surgery, lasting 14 days (*P* < 0.01, Figure 1); then, it increased to normal levels. To study the effects of TRPV4 and p38 with regard to neuropathic pain further, we sought to determine the abilities of RR, 4*α*-PDD, SB203580, and anisomycin to enhance or block the nociception signs induced 4 days after CCD surgery. As Figures 2(a) and 2(c) show, RR- and SB203580-treated rats exhibited paw withdrawals at higher mechanical forces compared with the saline group. In addition, 4*α*-PDD and anisomycin injections markedly decreased the paw withdrawal threshold compared with the saline group (Figures 5(b) and 5(d)). The most prominent time point was approximately 2 h after injection; no significant dose dependence was found.

### 3.2. Effects of Agonists and Inhibitors of TRPV4 and p38 on Protein Expression in CCD Rats

To investigate whether the TRPV4 and p38 expression changes affected each other, pharmacological agonists and inhibitors were given to CCD rats. Separately, the concentrations of these reagents were 1 nmol/L, 10 nmol/L, and 100 nmol/L for RR and 4*α*-PDD; 10 *μ*mol/L, 20 *μ*mol/L, and 40 *μ*mol/L for SB203580; and 5 *μ*g/mL, 25 *μ*g/mL, and 40 *μ*g/mL for anisomycin. The expressions of TRPV4, p38, and P-p38 were tested at 1, 2, 4, and 8 h after intrathecal injection of these drugs. The control group was given normal saline in an equal quantity.

**Figure 3:**
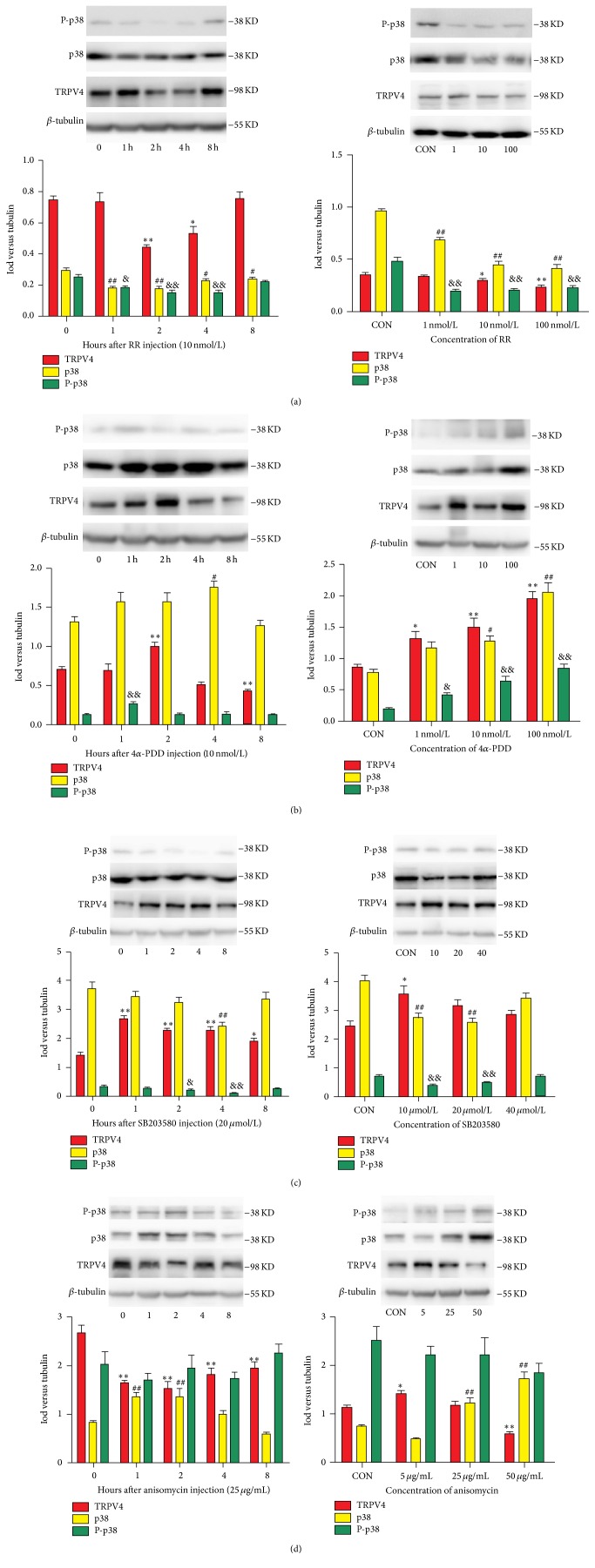
The effect of reagents on the protein expression of TRPV4, p38, and P-p38. (a–d) The expression of TRPV4, p38, and P-p38 proteins after RR, 4*α*-PDD, SB203580, and anisomycin intrathecal injections given to rats 4 days after CCD surgery. The bars represent means ± SEMs based on six experiments; ^*∗*^ *P* < 0.05 and ^*∗∗*^ *P* < 0.01, TRPV4 compared with controls. ^#^ *P* < 0.05 and ^##^ *P* < 0.01, p38 compared with controls. ^&^ *P* < 0.05 and ^&&^ *P* < 0.01, P-p38 compared with controls.

As shown in Figure 3(a), TRPV4, p38, and P-p38 were significantly inhibited by RR (2–4 h for TRPV4; 1–8 h for p38; and 1–4 h for P-p38), and both the TRPV4 and p38 changes were dose-dependent. When TRPV4 expression increased (see Figure 3(b)) by 4*α*-PDD (2 h), p38 and P-p38 were also upregulated (4 h for p38; 2 h for P-p38), and all compounds were associated with the concentration of the drug given. The administration of SB203580 (Figure 3(c)) significantly reduced the expression of p38 and P-p38 (4 h for p38; 2–4 h for P-p38) but significantly increased the expression of TRPV4 (1–8 h) regardless of the concentration. Finally, we gave anisomycin to the CCD rats (see Figure 3(d)); p38 protein expression was significantly increased (1-2 h), TRPV4 was inhibited (1–8 h), and P-p38 did not change significantly. A clear dose-dependent relationship was not found.

### 3.3. Protein Distribution Changes after Intrathecal Injections of TRPV4 and p38 Agonists and Inhibitors among CCD Rats

**Figure 4:**
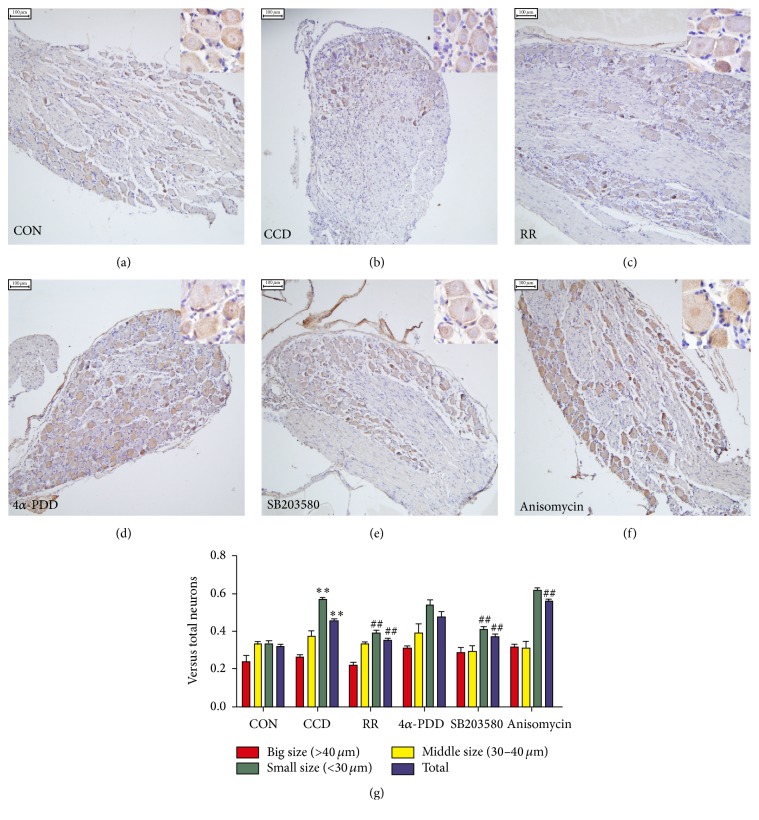
Altered distribution of TRPV4-positive neurons in DRG tissue. (a–f) TRPV4 immunohistochemical staining of the DRG neurons in the control, CCD, CCD+RR 10 nmol/L, CCD+4*α*-PDD 10 nmol/L, CCD+SB203580 20 *μ*mol/L, and CCD+anisomycin 25 *μ*g/mL groups, respectively. Scale bars: 100 *μ*m. (g) The analysis of the TRPV4-positive neurons (shown as means ± SEMs); ^*∗∗*^ *P* < 0.01 compared with controls; ^##^ *P* < 0.01 compared with the CCD group.

**Figure 5:**
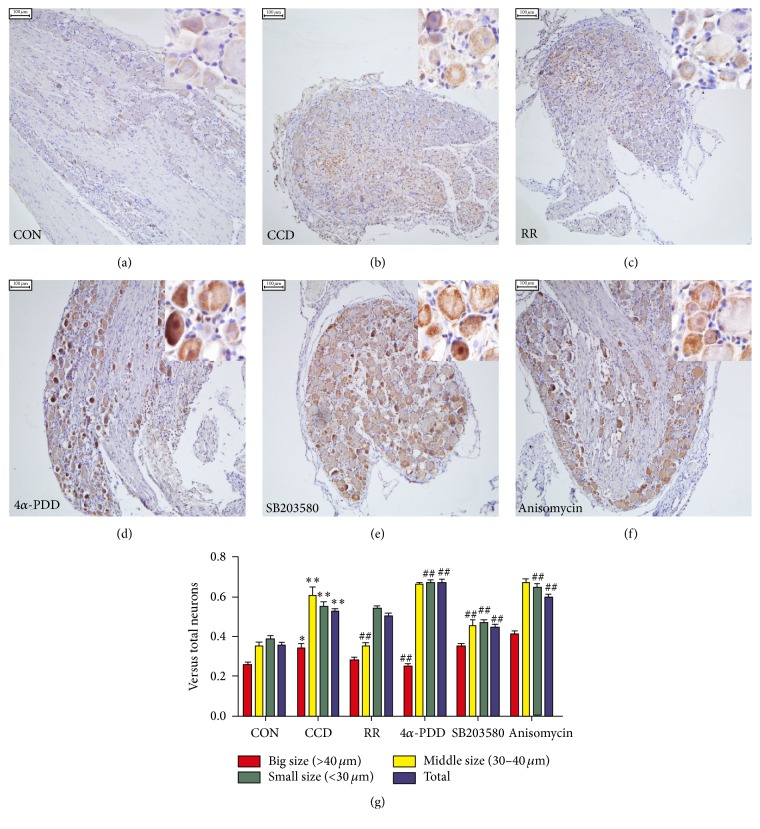
Distribution changes of the p38-positive neurons in DRG tissue. (a–f) p38 immunohistochemical staining of the DRG neurons in the control, CCD, CCD+RR 10 nmol/L, CCD+4*α*-PDD 10 nmol/L, CCD+SB203580 20 *μ*mol/L, and CCD+anisomycin 25 *μ*g/mL groups. Scale bars: 100 *μ*m. (g) The analysis of the p38-positive neurons (shown as means ± SEMs); ^*∗*^ *P* < 0.05 and ^*∗∗*^ *P* < 0.01 compared with controls; ^##^ *P* < 0.01 compared with the CCD group.

To evaluate whether the cellular distributions of TRPV4 and p38 within DRG neurons were altered because of CCD and the intrathecal injections of agonists and inhibitors, we used immunohistochemical staining to determine the proportion of TRPV4 and p38-positive neurons in the DRG tissues of CCD rats and controls after injection (Figures 4 and 5). We found that TRPV4 and p38 labeling were both evident in small, medium, and large ganglion cell bodies (small < 30 *μ*m, middle 30 *μ*m–40 *μ*m, and large > 40 *μ*m). The number of positive cells increased after CCD and was affected by the agonists and inhibitors. A quantitative analysis revealed that the number of TRPV4-positive neurons (Figure 4(g)) in the small and total ganglion neuron groups increased significantly (*P* < 0.01) compared with controls. Following the RR and SB203580 injections, the number of TRPV4-positive small neurons was reduced (*P* < 0.01). The total positive neuron number increased after anisomycin injection (*P* < 0.01), which significantly differed from the CCD group. As Figure 5(g) shows, the number of p38-positive neurons of all sizes was significantly increased after CCD compared with controls (*P* < 0.05, large; *P* < 0.01, medium, small, and total). The number of p38-positive, small neurons and the total number of p38-positive neurons were significantly reduced by SB203580 (*P* < 0.01) and increased by 4*α*-PDD (*P* < 0.01) and anisomycin (*P* < 0.01) compared with the CCD group.

### 3.4. The Effects of the Agonists and Inhibitors on Electrophysiological Properties

**Figure 6:**
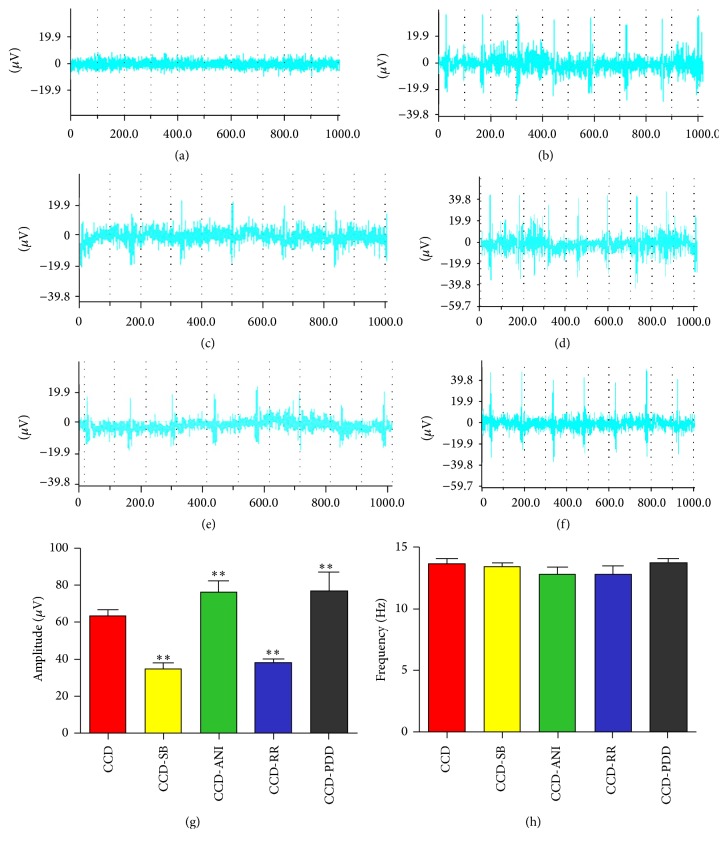
Ectopic discharges after CCD surgery and reagent injection. (a–f) represent discharges of the control, CCD, CCD+RR 10 nmol/L, CCD+4*α*-PDD 10 nmol/L, CCD+SB203580 20 *μ*mol/L, and CCD+anisomycin 25 *μ*g/mL groups. Cut from BL-420E+ biological and functional experimental system. (g) and (h) show the amplitudes and frequencies for the different groups (data are expressed as means ± SEM); ^*∗∗*^ *P* < 0.01, compared with the CCD group (7-8 rats in each group).

To confirm the contribution of TRPV4 and p38 with regard to spontaneous pain, we measured the ectopic discharges after CCD and the intrathecal injection of agonists or inhibitors. As Figure 6(a) shows, rare ectopic discharges occurred in normal rats. The frequencies of ectopic discharges did not markedly differ between groups (Figure 6(h)). However, the amplitudes (Figure 6(g)) in the RR and SB203580 groups were significantly reduced (*P* < 0.01) but significantly increased in the 4*α*-PDD and anisomycin groups (*P* < 0.01).

## 4. Discussion

The current study clearly shows that the expressions of TRPV4, p38, and P-p38 were elevated shortly after CCD surgery, whereas the PWMT decreased between 2 and 14 days after operation. We would like to evaluate rats at 4 days after CCD surgery in future experiments. When TRPV4 was activated by 4*α*-PDD, the expressions of TRPV4, p38, and P-p38 were upregulated and accompanied by increased TRPV4- and p38-positive small neurons in DRG tissue, anabatic mechanical allodynia, and larger amplitudes of ectopic discharges compared with the CCD rats given normal saline. The rats given RR injections exhibited opposite patterns of performance compared with those given 4*α*-PDD, though RR not only is a TRPV4 blocker but also can block other mechanically sensitive channels, as well as other channels that are not mechanically sensitive. We will try to find more TRPV4-specific blockers in the further studies. As p38 was activated in CCD rats due to anisomycin, the expression of p38 and P-p38 increased; however, anisomycin reduced TRPV4 expression. Interestingly, the number of TRPV4- and p38-positive small neurons in DRG tissue increased simultaneously and the amplitudes of ectopic discharges increased in CCD rats given anisomycin, similar to those given 4*α*-PDD. When the activation of p38 was inhibited by SB203580, the PWMT and ectopic discharges performed in the reverse manner among rats given anisomycin.

After CCD, the allodynia of rats and the hyperexcitability of their neurons were due to the activity of ion channels (e.g., voltage-gated Na+ and K+ channels), hyperpolarizing activated cation channels, and TRP channels. TRPV4 is a highly Ca^2+^-permeable cation channel found in the cell membrane, cytoplasm, and nucleus that plays a marked role in hyperalgesia [17]. p38 is the third MAPK found in mammals. This kinase is activated by intracellular [30] inflammatory cytokines (IL-1 and TNF-*α*) and cell stressors (UV, extracellular hypertonic solution, and heat shock) and takes part in intracellular inflammation and stress reactions as a single intracellular cascade reaction [33]. After DRG injury, the phosphorylation of p38 clearly increases, and SLN-induced transitory phosphorylation occurs 5 days after surgery [34]. The total protein expressions of TRPV4, p38, and P-p38 were tested after CCD, and the results showed that the chronic compression of DRG induced the upregulation of TRPV4 and p38 and facilitated the phosphorylation of p38; however, these changes were not completely synchronous with PWMT. These results reveal that TRPV4 and p38 are both involved in the mechanism of allodynia after CCD surgery, although other factors (e.g., the mechanism of central sensitization) should not be ignored.

When TRPV4 ion channels were activated or inhibited, the expression of p38 changed in parallel with TRPV4, and a similar result followed the phosphorylation of p38. These changes in the quantity and activation of TRPV4 alternated with the expression and phosphorylation of p38, which might be affected by the TRPV4-mediated calcium influx. When the agonist or inhibitor of p38 was given to CCD rats, the TRPV4 expression was the opposite of p38 expression, showing that the expression and phosphorylation of p38 affect TRPV4 through an unknown mechanism. Regarding the PWMT, TRPV4 and p38 agonists intensified allodynia after CCD, whereas TRPV4 and p38 inhibitors alleviated post-CCD allodynia, strongly demonstrating the function of TRPV4 and p38 in allodynia. Intrathecal injection is an effective method of intrathecal therapy, and the success rate of injection is more than 95%. Remarkably, the drugs are distributed not only to DRGs but also to the spinal cord. We would like to investigate the relationship between TRPV4 and p38 in the spinal dorsal horn and their effects on PWMTs in future studies.

As core components of the nervous system, neurons are involved in processing and transmitting signals, including inflammatory responses [35, 36]. These results show that the small neurons in the DRG might be the most important neurons for processing and transmitting pain signals. The L5-VRT model [37] demonstrated a method for investigating the role of uninjured neurons regarding neuropathic pain without sensory afferent injury. Eight to ten days after L5-VRT, the ipsilateral L4 and L5 DRGs of rats created stronger spontaneous discharges at the same time that mechanical hyperalgesia appeared. Of the two types of nociceptors, A*δ* for fast pain and C for slow pain, the excitability of C fibers changes with tissue injury and inflammatory stimuli, contributing to the formation of neuropathic pain. As demonstrated in the immunohistochemical experiments, the number of TRPV4- or p38-positive small neurons changed significantly after CCD and was alternated by the agonists and inhibitors of TRPV4 and p38. Thus, the small neurons branching out into C fibers might be the most important neurons for neuropathic pain. DRGs are the primary neurons for sensory afferents, primarily nourishing and supporting the nerves that branch from them. Under normal conditions, DRG neuron bodies do not transport electoral signals directly nor do they create spontaneous discharges [38]. After DRG injuries, the physiological function of the neurons changes, and they become unduly excited. Thus, slight chemical or physical stimuli can induce large amounts of ectopic discharges from neurons* in vivo*, making them a signal source and leading to neuropathic pain that presents as spontaneous pain, hyperalgesia, and allodynia. The results of electrophysiological experiments showed that the ectopic discharges appeared after CCD, and the amplitudes were affected by activated or inhibited TRPV4 and p38, similar to the changes in small neurons. The small neurons take part in the formation of ectopic discharges mediated by TRPV4 and p38, and the TRPV4-p38 pathway might be one mechanism of ectopic discharge.

## 5. Conclusion

In conclusion, the expression and activation of TRPV4 and p38 change after CCD significantly contribute to the alternation of PWMT and the amplitudes of ectopic discharges, acting upon each other, mostly in small neurons. The current studies provide evidence for the existence of a link between TRPV4 and p38, with an intermediary role for neuropathic pain. Furthermore, the link between TRPV4 and p38 is bidirectional.
